# Integrated genomics, metagenomics and metatranscriptomics to reveal the biocontrol mechanism of *Bacillus velezensis* JY10 against tobacco target spot disease

**DOI:** 10.3389/fmicb.2025.1707097

**Published:** 2025-12-08

**Authors:** Wenjuan Yang, Hui Liu, Rubing Xu, Yiqian Peng, Tingting Xu, Yong Yang, Yanyan Li, Haibo Xiang

**Affiliations:** 1 College of Life Sciences, Hubei University, Wuhan, China; 2 Hubei Provincial Key Laboratory of Occurrence and Intervention of Kidney Diseases, Hubei Provincial Engineering Research Center of Immunotherapy Drugs for Renal Tumors, School of Medicine, Hubei Polytechnic University, Huangshi, China; 3 Tobacco Research Institute of Hubei Province, Wuhan, China; 4 College of Plant Science and Technology, Huazhong Agricultural University, Wuhan, China

**Keywords:** tobacco target spot disease, *Rhizoctonia solani*, *Bacillus velezensis*, genomics, metagenomics, metatranscriptomics

## Abstract

Tobacco target spot (TTS) disease, a prevalent fungal disease caused by *Rhizoctonia solani*, severely reduces tobacco yield and quality, imposing substantial economic losses on the tobacco industry. In this study, we employed a biological control approach against TTS using a *Bacillus velezensis* JY10 isolated from healthy tobacco stems. We further elucidated the mechanism of JY10 in controlling TTS through genomics, metagenomics and metatranscriptomics. The results showed that JY10 exhibited robust inhibitory effects against *R. solani*, with an inhibition rate exceeding 95%, and achieved a TTS control efficacy of 68.63% in pot experiments. Whole-genome sequencing demonstrated that the JY10 genome spans 3,929,772 bp, contains 4,026 protein-coding genes, and has a GC content of 46.5%. AntiSMASH analysis predicted 12 secondary metabolite biosynthetic gene clusters, encoding antimicrobial compounds such as surfactin, fengycin, difficidin, bacillaene, bacillibactin, macrolactin H, and bacilysin. Metagenomic profiling showed that JY10 treatment had no significant influence on tobacco phyllosphere and rhizosphere microbiome structure, however, it significantly increased the relative abundance of beneficial microbes, including *Bacillus, Pseudonocardia, and Pseudomonas*. Metatranscriptomic analysis revealed that JY10 might enhance tobacco TTS resistance by modulating oxidative phosphorylation pathway and upregulating several antibiotics biosynthesis. Taken together, JY10 may employ a dual control strategy against TTS, involving the direct production of antifungal compounds, as well as indirectly increasing the abundance of beneficial microbes and modulating their oxidative phosphorylation and antibiotic synthesis pathways in the phyllosphere and rhizosphere of tobacco. These findings provide a theoretical foundation for understanding biocontrol mechanisms of JY10 and introduce a promising bacterial resource for the development of sustainable TTS management strategies.

## 1 Introduction

Tobacco (*Nicotiana tabacum* L.) target spot disease (TTS) is a leaf disease in tobacco caused by *Rhizoctonia solani* Kühn (sexual form: *Thanatephorus cucumeris* (Frank) Donk), a pathogen widely existing in soil (Gonzalez et al., 2011; Tang et al., 2023). It can infect plants from the seedling stage to maturity. In the early stages of the disease, infected tobacco leaves exhibit small, round lesions resembling water stains, with a diameter of about 2–3 mm. As the disease progresses, these lesions develop into irregular shapes, increasing in size to 2–3 cm. Yellow green halos with irregular concentric rings can be observed around the yellow brown spots on the edges. In the late stage of the disease, both the center and edges of the lesions are prone to tearing, leading to perforations in the leaves, which resemble bullet holes in a target, hence the common name “target spot” disease (Chacon et al., 2010; Sun et al., 2023). TTS is prevalent in many countries, resulting in decreased tobacco production and quality, which leads to significant economic losses (Ahsan et al., 2017; Mercado Cárdenas et al., 2012; Zhong et al., 2023). In southwest China, losses can exceed 50% without effective disease management (Sun et al., 2023). Furthermore, *R. solani* is responsible for other severe agricultural diseases, including rice sheath wilt, pepper root rot, peanut pod rot, tomato root rot, and wheat root rot, all of which cause substantial losses worldwide (Huang et al., 2024). Therefore, it is urgent to develop management strategies to control *R. solani*.

Currently, the management of TTS disease predominantly relies on chemical agents. For instance, iprodione and pyraclostrobin are recognized as effective chemical agents for controlling this disease (Csinos and Stephenson, 1999; Yin et al., 2024). However, the prolonged application of chemical pesticides can result in environmental pollution and pose risks to both human and animal health, which contradicts the principles of green and sustainable development. In contrast, biological control offers advantages such as being nontoxic, residue-free, and environmentally friendly (Yang et al., 2024). Therefore, it is essential to screen antagonistic microorganisms with biocontrol potential against the pathogens responsible for TTS disease and to provide microbial resources for its biological control. This approach is crucial for reducing dependence on chemical pesticides and promoting the green and sustainable development of tobacco.

*Bacillus* spp. has been extensively studied for their effective control activity against *R. solani* (Zhu et al., 2023; Huang et al., 2024; Ali et al., 2025; Marzouk et al., 2025). In particular, *Bacillus velezensis* has attracted researchers’ attention due to its promising results in controlling destructive phytopathogens in field and greenhouse crop cultivation, as well as in biofungicide manufacturing (Keshmirshekan et al., 2024). It has been found that *B. velezensis* exhibits potent inhibitory effects against various *R. solani* separated from different kinds of diseased crops, with mycelial inhibition rates of up to 77.33% (Alam et al., 2025; Huang et al., 2023; Naveena et al., 2025). Furthermore, it demonstrates high control efficiency (up to 60%) against rice sheath blight, potato stem canker and black scurf diseases, bean root rot, and cotton rhizoctonia damping-off caused by *R. solani* (Lee et al., 2025; Su et al., 2023; Tao et al., 2024; Wekesa et al., 2023). While these reports primarily focus on the inhibitory effects of antagonistic microbes on pathogenic fungi, few studies have explored the disease resistance mechanisms of these antagonistic microbes and their impact on the phyllosphere and rhizosphere microbiomes of host plants. This study identified a *Bacillus* strain, JY10, with disease resistance capabilities from tobacco. Through genomic, metagenomic, and metatranscriptomic sequencing analyses, we investigated the changes in the phyllosphere and rhizosphere microbial communities of tobacco under JY10 treatment, as well as the differential gene expression. The aim of this study is to provide a theoretical foundation and a new biocontrol resource for the management of TTS disease by evaluating the biocontrol potential of JY10 against TTS and exploring its underlying biocontrol mechanisms.

## 2 Materials and methods

### 2.1 Pathogen strains and plant seeds

The pathogens *R. solani, Colletotrichum fructicola*, and *Boeremia linicola*, and tobacco seeds of Yunyan87 were provided by Tobacco Research Institute of Hubei Province. *R. solani, C. fructicola*, and *B. linicola* were cultured on PDA medium (200 g/L potato peels, 20 g/L dextrose, 15 g/L agar, pH 7) at 28 °C. The bacteria were cultured on NA medium (glucose: 10 g/L, peptone: 5 g/L, beef extract: 3 g /L, yeast extract: 3 g/L, 15 g/L agar, pH 7) or LB medium (peptone: 10 g/L, NaCl: 10 g /L, yeast extract: 5 g/L) at 30 °C. The tobacco seeds were sown in floating polystyrene trays in a greenhouse with 16/8 h day/night cycle at 26°C.

### 2.2 Isolation, screening of antagonistic bacteria against *R. solani* and resistance determination of strain JY10 to other tobacco leaf pathogens

The healthy tobacco plants were collected in the tobacco planting fields (29.99°N, 109.44°E) in Xuanen County, Enshi City, Hubei Province. The plants were rinsed with tap water and subjected to surface sterilization with 75% ethanol and 2% sodium hypochlorite (root: 5+3 min; stem 4+2 min; leaf: 2+2 min), followed by rinsing with sterile distilled water. The different plant tissues were then ground 6–8 times in a rapid grinder at a speed of 60 Hz and at a frequency of 60 times per minute. The ground tissue suspension was spread onto the NA medium and placed in a 30°C constant temperature incubator for 2–4 days. Then, single colonies with different shapes, sizes or colors were picked and streaked onto NA slants until pure cultures were obtained.

The plate confrontation method was used to screen antagonistic bacteria against *R. solani* and determine the inhibitory ability of JY10 against*C. fructicola* and*B. linicola* (Zhu et al., 2025). Briefly, the endophytic bacteria were streaked centrally on a PDA plate, with the pathogen patches inoculated on both sides. After incubation, the inhibition zone width was measured against a pathogen-only control once the control mycelia reached the plate edge. Each treatment was repeated at least thrice.

### 2.3 Pot experiments

Pot experiments were conducted in a greenhouse at Hubei University using a randomized block design. Each treatment consisted of six individual plants, and the experiment was repeated three times. Tobacco seedlings that grew to the 4/5-leaf stage were randomly divided into three groups: CK1, which received a spray of sterile water; CK2, which received a spray of sterile water followed by the inoculation of *R. solani* (5 mm in diameter) onto the 3rd leaf of each tobacco plant 24 h later; and JY10, which was sprayed with JY10 (1 × 10^8^ CFU/mL) followed by the inoculation of *R. solani*. The mycelial plug was removed 24 h after inoculation, while maintaining humidity throughout the inoculation period. Three days after inoculation, the lesion diameter of tobacco leaves was measured, and the lesion area and control efficiency were calculated with the formulars below: *LA* = *L* × *W*, *CE* = (*LA*_CK2_ – *LA*_T_/*LA*_CK2_ × 100%, where *LA* represents lesion area, *L* and *W* represent the length and width of lesion area, which were measured by cross-measurement method. *CE* representss control efficiency, *LA*_*CK*2_ and *LA*_*T*_ represent the lesion area of CK2 group and JY10 group, respectively.

### 2.4 Morphological observation and physiobiochemical tests of strain JY10

The morphological observation and Gram staining of strain JY10 were examined as described by Zhu et al. (2023). The physiobiochemical characteristics of JY10 were determined according to Bergey’s Manual of Systematic Bacteriology (Volume 4) (Whitman, 2010). The test contents included carbon source utilization (D-sucrose, D-maltose, D-galactose, α-lactose), acid production (D-glucose, D-xylose, L-arabinose, D-mannitol), citrate utilization, methyl red test, urea hydrolysis, and negative results of phenylalanine dehydrogenase experiment; catalase test, V-P test, starch hydrolysis, gelatin hydrolysis, and nitrate reduction reaction.

### 2.5 Genome sequencing and analysis

Genomic DNA of JY10 was extracted with a commercial DNA extraction kit (Thermofisher, United States). The harvested DNA was detected by the agarose gel electrophoresis and quantified by Qubit^®^ 2.0 Fluorometer (Thermo Scientific, United States). Genome sequencing was performed with Nanopore PromethION platform and Illumina NovaSeq PE150 platform in Beijing Novagene company. The PE150 raw data and Nanopore raw data were assembled to no gap complete sequence with Unicycler software. GeneMarkS (Version 4.17) was used to predict the coding genes. The biosynthetic gene clusters of antimicrobial compounds were mining with antiSMASH.

### 2.6 Phylogenetic analysis

The genomes of 17 *Bacillus* and *Mycobacterium tuberculosis strain CDC1551* were downloaded from Genebank database, and the core- and pan-genes of these strains were analyzed by the CD-HIT rapid clustering of similar proteins software with a threshold of 50% pairwise identity and 0.7 length difference cutoff in amino acid. Then, a maximum likelihood phylogenetic tree was constructed with TreeBeST based on 560 core genes shared by these strains and the setting of bootstraps was 1,000 with the orthologous genes. The average nucleotide identity (ANI) between the whole-genome sequences was calculated using JspeciesWS^1^ and the heatmap was drawn with CIMminer (Gu et al., 2021).

### 2.7 Metagenomic and metatranscriptomic sequencing and analysis

Tobacco plants at the four-leaf stage were subjected to foliar spraying with JY10 at a concentration of 1 × 10^8^ CFU/mL. Leaves and rhizosphere soil were harvested 72 h post-treatment, and DNA, RNA and cDNAs were extracted and synthesized using DNA/RNA extraction kits and cDNA synthesis kit (Thermofisher, United States). Metagenomic and metatranscriptomic sequencing was performed at Novogene Bioinformatics Technology Co., Ltd. (Beijing, China) using Illumina HiSeq PE150 platform. Each treatment group involved three biological replicates, and each replicate was sequenced and data analyzed separately.

Readfq^2^ was used for preprocessing raw data of metagenomic sequencing from the Illumina sequencing platform to obtain clean data. Subsequently, low quality nucleotides, any read with N bases, and potential host-derived sequences were discarded with fastp^3^ and Bowtie2 software.^4^ MEGAHIT software^5^ was used for assembly analysis of clean data, and MetaGeneMark^6^ was used to perform ORF prediction. For the ORF prediction results, CD-HIT software^7^ was used to eliminate redundancy and obtain the non-redundant initial gene catalogue. To perform taxonomic and functional analysis, the genes were compared (BLASTp) against NCBI-nr,^8^ COG,^9^ KEGG,^10^ eggnog,^11^ and CAZy database^12^ using DIAMOND software^13^ with an *e*-value cutoff of 10^–5^ (Yan et al., 2024; Zheng et al., 2024). LEfSe (v1.1.2) were used for screening of key differentially abundant species.

The raw reads of metatranscriptomic sequencing were firstly processed through fastp software to obtain clean data. Next, de novo assembly was performed with Trinity,^14^ and the sequence of all samples was integrated and repeat (set sequence identity threshold 0.95) was deleted using CORSET^15^ to obtain unigene dataset. Then, DIAMOND software was used to compare unigenes with the sequences of bacteria, fungi, archaea and viruses extracted from NCBI’s NR database (blastp) with an *e*-value cutoff of 10^–5^. After filtering, reads that match the genes sequence in the NR database, were placed to the lowest common ancestor (LCA) node of those species in the taxonomy that were known to have that gene (LCA algorithm, applied to the system classification of MEGAN software) (Huson et al., 2011). The classification level before the first branch would appear as the species annotation information of the sequence. Gene function annotations were performed by searching genes against four major databases (GO, KEGG, CAZy and eggNOG) using DIAMOND software with an *e*-value cutoff of 10^–5^. Quantification of the transcripts and genes was performed with the fragments per kilobase of transcript sequence per millions base pairs (FPKM)-normalization method using RSEM (Li and Dewey, 2011). DESeq2 was used for differential expression analysis. The resulting *p*-values were adjusted using the Benjamini and Hochberg’s approach for controlling the false discovery rate. Genes with | log2 (Fold Change) | > 1 and *p*adj < 0.05 were assigned as differentially expressed.

### 2.8 qRT-PCR analysis

For the validation of metatranscriptomic results, some genes involved in antibiotic synthesis were verified via quantitative reverse transcription PCR (qRT-PCR) with three biological replicates following the method described by Medina Munoz et al. (2021). Briefly, metatranscriptomic RNAs were reverse transcription into single-stranded cDNA using PrimeScript™ II 1st Strand cDNA Synthesis Kit (Takara, Japan). Linearized pGEM^®^-T plasmid standards were used as spike-in control for the quantification of gene copy numbers. Primers for the selected transcript genes (Supplementary Table 1) were designed with Primer Premier 6.0. Quantitative PCR was conducted using the Bio-Rad CFX96 Real-Time System with PowerUP SYBR Green Master Mix (Applied Biosystems). The amplification conditions included denaturation at 95 °C for 2 min, followed by 40 cycles of denaturation at 95 °C for 15 s, annealing at 55 °C for 15 s, and extension at 72 °C for 50 s. The relative gene expression was determined using the 2^– ΔΔCt^ method.

### 2.9 Statistical analysis

All data were analyzed using the one-way analysis of variance (ANOVA) method with SPSS 22.0 software (SPSS Inc., Chicago, United States) and Duncan’s multiple range test was used to test the significance of the difference between treatments (*P* < 0.05). PCoA was performed to ordinate the microbial composition in the different samples based on Bray-Curtis distance with the vegan and ggplot2 packages in the R software. Permutational multivariate analysis of variance (PERMANOVA) was performed with 999 permutations through the adonis function implemented in vegan package.

## 3 Results

### 3.1 Screening of endophytic bacteria antagonizing *R. solani*

**FIGURE 1:**
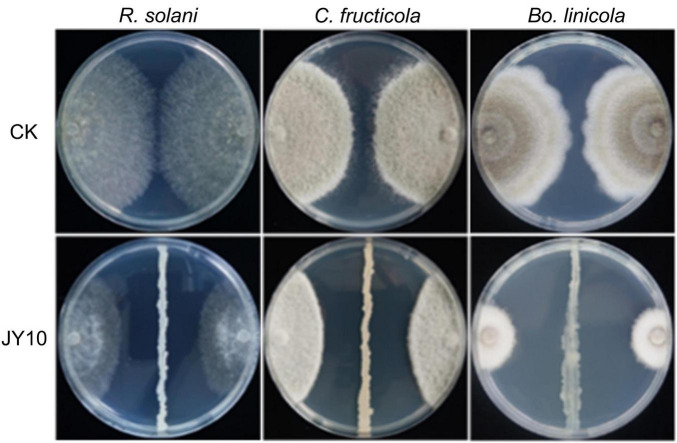
Inhibitory effects of strain JY10 on *R. solani, C. fructicola* and *B. linicola.*

A total of 70 endophytic bacteria were isolated from healthy tobacco plants. Among these, 48 strains were isolated from the roots, 12 strains from the leaves, and 10 strains from the stems. Through antagonistic activity assays performed with three biological replicates, each including technical replicates, 19 bacteria strains exhibited potent inhibitory effects (diameter of inhibition zone > 10 mm) on *R. solani*, among which JY10 had the highest antagonistic activity with an inhibition zone diameter of 17.58 mm (*p* = 2.89e-6) (Figure 1, Supplementary Figure 1, and Supplementary Table 2).

In addition to *R. solani*, JY10 also displayed strong inhibitory effects against *C. fructicola* and *B. linicola*, which are responsible for tobacco anthracnose and tobacco *Phoma* leaf spot disease, respectively. The inhibition zone diameters against *C. fructicola* and *B. linicola* reached 18.58 mm and 24.95 mm (Figure 1), respectively, suggesting that JY10 possesses broad-spectrum antagonistic activity against fungal pathogens that cause various tobacco leaf diseases.

### 3.2 The greenhouse control effect of JY10 on TTS

**FIGURE 2:**
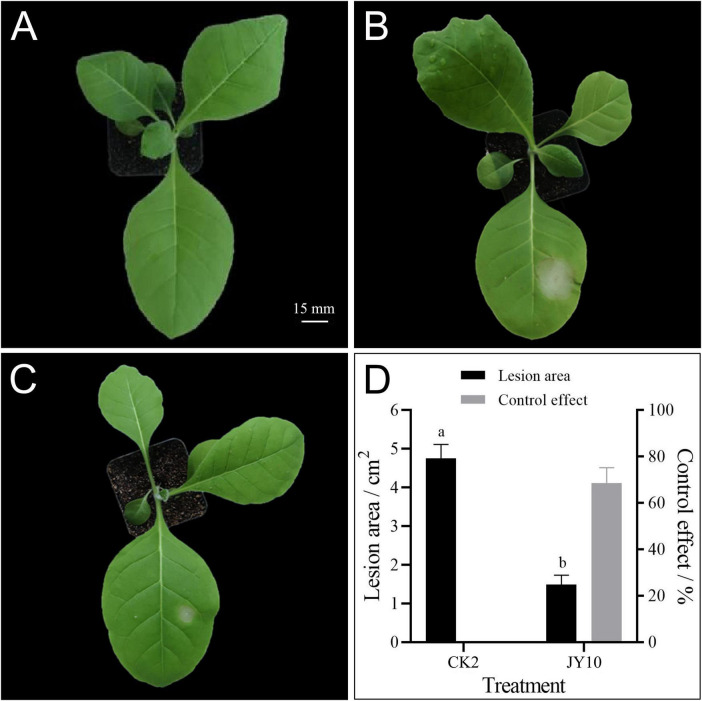
Potted control effect of JY10 on TTS disease. Healthy tobacco seedlings were treated with sterile water as positive control **(A)**, *R. solani* as negative control **(B)**, and JY10 followed by *R. solani* **(C)**. The lesion areas and control effects of treatments were calculated 3 days after inoculation **(D)**.

To further confirm the control effect of JY10 against TTS caused by *R. solani* in planta, we conducted a greenhouse experiment. The results of the pot experiment indicated that, compared to the control group (CK), tobacco leaves inoculated with R. solani exhibited severe TTS disease symptoms, with the lesion area measuring 4.75 ± 0.35 cm^2^. However, when JY10 was sprayed 24 h prior to the inoculation of *R. solani*, the severity of TTS disease symptoms was significantly reduced, resulting in a lesion area of 1.49 ± 0.24 cm^2^, which corresponded to a control efficacy of 68.63% (*p* = 4.34e-4) (Figure 2). This finding suggested that JY10 is effective in controlling TTS disease.

### 3.3 Morphological and physiobiochemical characteristics of JY10

**FIGURE 3:**
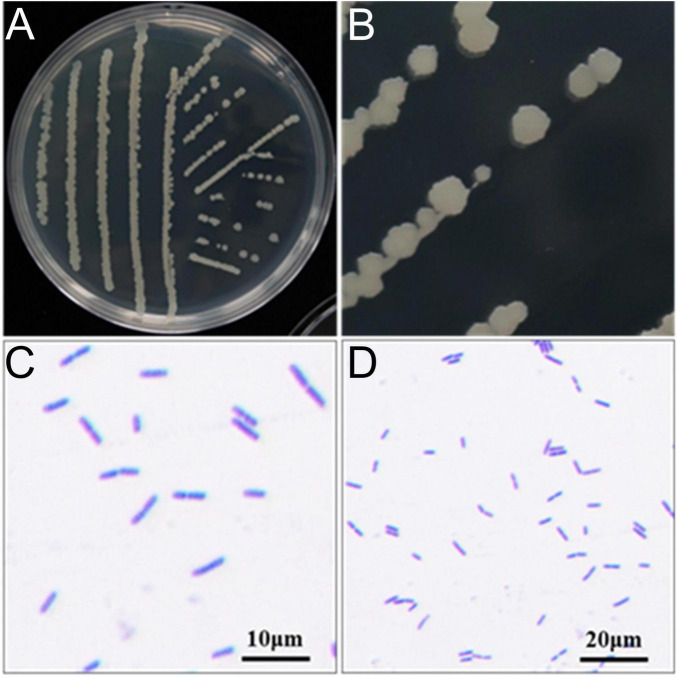
The morphological characteristics **(A,B)** and Gram staining **(C,D)** of strain JY10.

Next, we identified JY10 through morphological observation and physiobiochemical experiments. After being cultured on LB solid medium for 24 h, the colony of strain JY10 turned to a light yellow color, with a smooth and flat surface and neat edges (Figures 3A,B). The Gram staining result was positive, and the bacterial cells were rod-shaped (Figures 3C,D).

The results of the physiological and biochemical experiments indicated that JY10 could utilize various carbon sources including D-sucrose, D-maltose, D-galactose, α-lactose, but could not utilize D-fructose. Additionally, it was capable of producing acid from D-glucose, D-xylulose, L-arabinose, and D-mannitol. The tests for citrate utilization, methyl red test, urea hydrolysis, and phenylalanine dehydrogenase were negative. Conversely, the catalase reaction, V-P test, starch hydrolysis, gelatin hydrolysis, and nitrate reduction yielded positive results (Table 1 and Supplementary Figure 2). Based on the morphological and physiobiochemical characteristics, the strain JY10 was identified as *Bacillus*.

**TABLE 1:** Physiological and biochemical characteristics of JY10.

Physiological and biochemical item	JY10
D-sucrose utilization	+
D-fructose utilization	-
D-maltose utilization	+
D-galactose utilization	+
α-lactose utilization	+
Citrate utilization	-
Contact enzyme reaction	+
D-glucose acid production	+
D-xylose acid production	+
L-arabinose acid production	+
D-mannitol acid production	+
Methyl red test	-
Voges-Proskauer test	+
Starch hydrolysis	+
Gelatin hydrolysis	+
Urea hydrolysis	-
Nitrate reduction	+
Phenylalanine dehydrogenase	-

### 3.4 Genomic features of JY10

Furthermore, we conducted whole-genome sequencing of JY10 to further identify it and explore its control mechanism against TTS. The results of the high-throughput sequencing revealed that the JY10 genome comprises a single circular chromosome with a size of 3,929,772 bp, a GC content of 46.5%, and a total of 4,026 protein-coding genes (Table 2; Supplementary Figure 3). The complete genome data of JY10 have been deposited in GenBank under the accession number SRR31156179.

**TABLE 2:** General genome features of JY10.

Category	JY10
Genome size (bp)	3,929,772
GC content (%)	46.5
Protein-coding number	4026
Gene total length (bp)	3,526,641
Gene average length (bp)	876
tRNA	86
rRNA	9

### 3.5 Phylogenetic analysis of JY10

**FIGURE 4:**
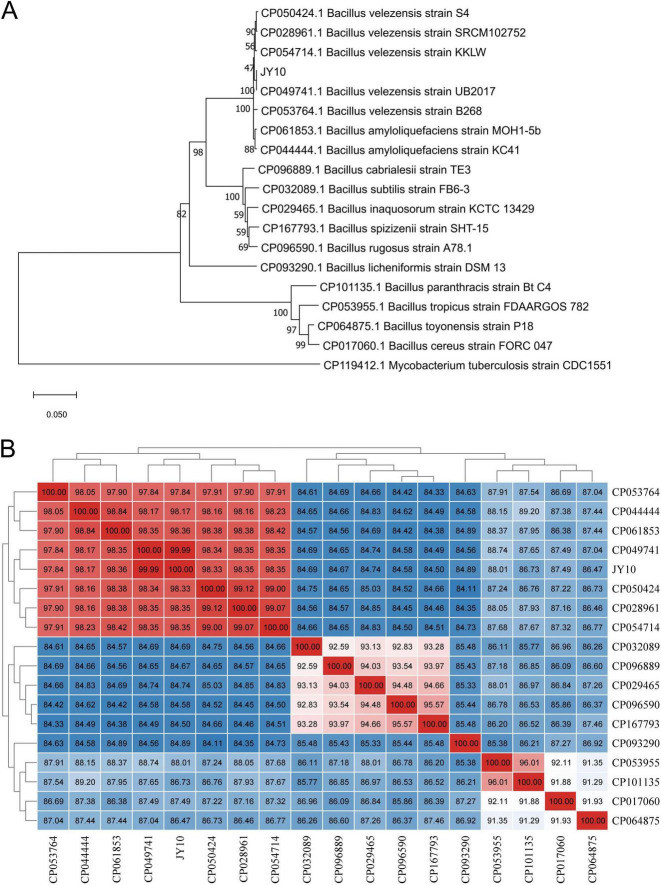
Phylogenetical analysis of JY10. Phylogenetic tree constructed based on the 560 single-copy core genes **(A)**, and heat-map of ANI values amongst different *Bacillus* strains **(B)**. The numbers represent the size of ANI values.

Based on the genomic features above, we compared the pan- and core-genome sizes between JY10 and 17 other *Bacillus* species. The result indicated that the pan-genome consisted of 560 core genes, while JY10 possessed 3739 species-specific genes, surpassing *Bacillus velezensis* B268 (3683), *Bacillus velezensis* KKLW (3717) and *Bacillus amyloliquefaciens* MOH1-5b (3727) (Supplementary Table 3). A phylogenetic tree of JY10 and the 17 *Bacillus* was constructed based on the concatenation of 560 single-copy core genes present in all genomes utilizing the maximum likelihood (ML) method and rooted in *Mycobacterium tuberculosis strain CDC1551*. As shown in Figure 4A, strain JY10 is located in the same clade with other *Bacillus velezensis* strains and is a sister group of *B*. *velezensis* UB2017. Subsequently, we conducted a heatmap analysis based on the average nucleotide identity (ANI) values of different strains to corroborate the findings of our phylogenetic analysis. As illustrated in Figure 3B, JY10 and four *B*. *velezensis* strains (*B*. *velezensis* S4, *B*. *velezensis* SRCM102752, *B*. *velezensis* KKLW and *B*. *velezensis* UB2017) showed ANI values of > 98.3%, of which *B*. *velezensis* UB2017 exhibited highest ANI values to JY10 (99.99%). Based on the morphological, physiobiochemical and genomic characteristics, strain JY10 was identified as *B*. *velezensis*.

### 3.6 Genetic basis for the anti-pathogen activity of JY10

Subsequently, we analyzed the secondary metabolite synthesis gene cluster of JY10 genome with antiSmash. AntiSmash analysis predicted 12 biosynthetic gene clusters (BGCs) of 8 types, including betalactone, PKS-like, T3PKS, transAT-PKS, NRPS, RiPP-like, terpene, and lanthipeptide-class-II (Table 3). The genome of JY10 contains gene clusters responsible for the production of several antifungal compounds, such as surfactin, fengycin, and bacillibactin, the latter of which also exhibits antibacterial and iron-scavenging activities. Furthermore, the JY10 genome encodes antibacterial compounds, including butirosin A/B, macrolactin H, bacillaene, difficidin, and bacilysin.

**TABLE 3:** List of putative secondary metabolite producing biosynthetic clusters of JY10 as predicted by antiSMASH.

Type	BGC length (bp)	Most similar known cluster	MIBiG ID	Similarity	Activitiy
NRPS	65,407	Surfactin	BGC0000433	86%	Anti-bacteria, anti-fungi, antimycoplasma
PKS-like	41,244	Butirosin A/B	BGC0000693	7%	Anti-bacteria
Terpene	20,740
Lanthipeptide-class-ii	28,888
Transat-PKS	88,233	Macrolactin H	BGC0000181	100%	Anti-bacteria
Transat-PKS, T3PKS, NRPS	110,105	Bacillaene	BGC0001089	100%	Anti-bacteria
NRPS, transAT-PKS, betalactone	137,801	Fengycin	BGC0001095	100%	Antifungal
Terpene	21,883
T3PKS	41,106
Transat-PKS	106,182	Difficidin		100%	Anti-bacteria
NRP-metallophore, NRPS, RiPP-like	51,791	Bacillibactin	BGC0000309	100%	Antibiotic, Fe-chelating molecule
Other	41,418	Bacilysin	BGC0001184	100%	Anti-bacteria

### 3.7 Changes in microbiome of tobacco phyllosphere and rhizosphere after treatment with JY10

**FIGURE 5:**
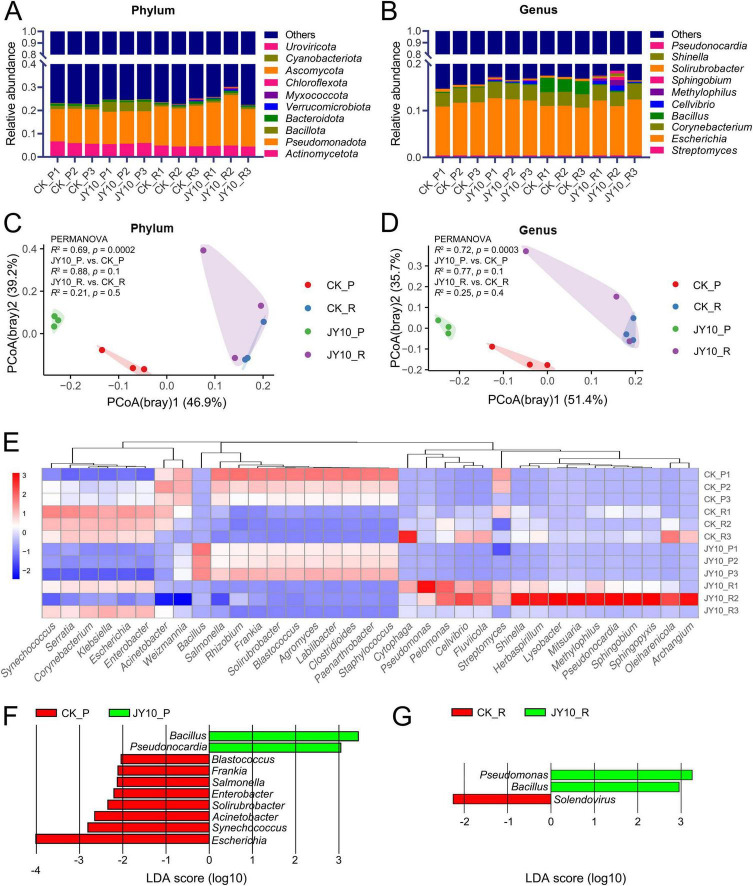
Changes in microbiome of tobacco phyllosphere and rhizosphere after treatment with JY10. Microbial community composition at the phylum **(A)** and genus levels **(B)**; PCoA of the microbial community at the phylum **(C)** and genus levels **(D)**; Heatmap of the relative abundance of 35 microbial genera **(E)**; Distribution map of linear discriminant analysis (LDA), values for different genus in leaf **(F)** and rhizosphere **(G)** (*P* < 0.05). P: phyllosphere, R: rhizosphere.

We further explored the TTS resistance mechanisms of JY10 in terms of its impact on the phyllosphere and rhizosphere microbiomes of host plants through metagenomics. The metagenomic sequencing generated a total of 179.16 Gbp of raw reads and 178.46 Gbp of clean data after quality control (Supplementary Table 4). The assembly results revealed an average of 359,146 scaftigs per sample, with an average N50 and N90 length of 679 and 527 bp, respectively. The raw data were deposited in NCBI with bioproject submission number SUB15467664. A total of 4.87 million non-redundant genes were identified, with an average open reading frame (ORF) length of 294 bp, suggesting high sequencing quality. The operational taxonomic units (OTUs), Chao1 and Shannon index were used to evaluate and compare the diversity and richness of microbial community among different treatment groups (Table 4). Compared to the control group, the OTUs, Chao1 and Shannon index for the phyllosphere and rhizosphere treated with JY10 exhibited increases, although these differences were not statistically significant (The *p*-values for OTUs, Chao1 and Shannon index of phyllosphere were 0.73, 0.62 and 0.80, and of rhizosphere were 0.41, 0.46 and 0.18, respectively). This suggested that JY10 treatment had a limited impact on the microbial community diversity of tobacco plants. The top 10 abundant microbial phyla and genera were selected to compare the changes of microbial communities in phyllosphere and rhizosphere under different treatments (Figures 5A,B; Supplementary Tables 5, 6). The phyla Pseudomonadota, Actinomycetota, Bacillota, Bacteroidota, Cyanobacteriota, Ascomycota, Myxococcota, Chloroflexota, Verrucomicrobiota, and Uroviricota were identified as the most abundant. Notably, the relative abundances of Bacillota and Uroviricota in phyllosphere increased three-fold following JY10 treatment, while no significant changes were observed in the rhizosphere. Additionally, the genera *Escherichia, Corynebacterium, Solirubrobacter, Streptomyces, Bacillus, Pseudonocardia, Methylophilus, Sphingobium, Shinella*, and *Cellvibrio* were the top 10 abundant genera, with *Bacillus* in the phyllosphere showing a significant increase of 12-fold. At both the phylum and genus levels, principal coordinates analysis (PCoA) based on the Bray-Curtis distance showed significant differences in microbial community structure of both the phyllosphere (PERMANOVA, *R*^2^ = 0.69, *p* = 0.0002) and rhizosphere (PERMANOVA, *R*^2^ = 0.69, *p* = 0.0002) among the four treatment groups (Figures 5C,D). However, when conducting pairwise comparisons, this significant difference dissipated (*p* > 0.05), which was consistent with the alpha-diversity results. This finding suggested that the apparent significant differences observed among the four groups arised from the overall significance resulting from the aggregation of multiple groups of minor differences, rather than a substantial difference between any two groups.

**TABLE 4:** Alpha diversity index of microbiome of tobacco phyllosphere (P) and rhizosphere (R) at 3 d post-treatment based on metagenomic data.

Treatment	OTUs	Chao1	Shannon
CK_P	1186 ± 6.68b	1203.07 ± 7.13b	2.33 ± 0.09a
CK_R	2244 ± 166.43a	2204.36 ± 69.79a	2.30 ± 0.15a
JY10_P	1229 ± 28.96b	1258.11 ± 37.15b	2.39 ± 0.05a
JY10_R	2349 ± 172.65a	2490.32 ± 35.08a	2.62 ± 0.39a

The different letters in the same column indicate significant differences at *p* < 0.05 according to LSD test.

Additionally, a heatmap analysis of the top 35 genera, arranged in hierarchical clusters, was conducted to elucidate the differing compositions of microbial community structures (Figure 5E). The relative abundance of *Bacillus* and *Sphingopyxis* were significant higher in the phyllosphere of the JY10_vs._CK group, whereas *Acinetobacter* and *Weizmannia* exhibited markedly lower abundances. No significant changes were noted in the rhizosphere of the JY10_vs._CK group. Comparative LEfSe analyses (LDA score > 2.5) further highlighted that *Bacillus* and *Pseudonocardia* were significantly enriched in the phyllosphere of the JY10 group, while the CK group exhibited a greater diversity of microbial species, including *Blastococcus*, *Frankia*, *Salmonella, Enterobacter, Solirubrobacter, Acinetobacter, Synechococcus*, and *Escherichia* (Figure 5F). In the rhizosphere, *Bacillus* and *Pseudomonas* from the JY10 group, along with *Solendovirus* from the CK group were identified to have a greater influence on the differences observed (Figure 5G).

### 3.8 Metatranscriptomic analysis of tobacco plants treated with JY10

**FIGURE 6:**
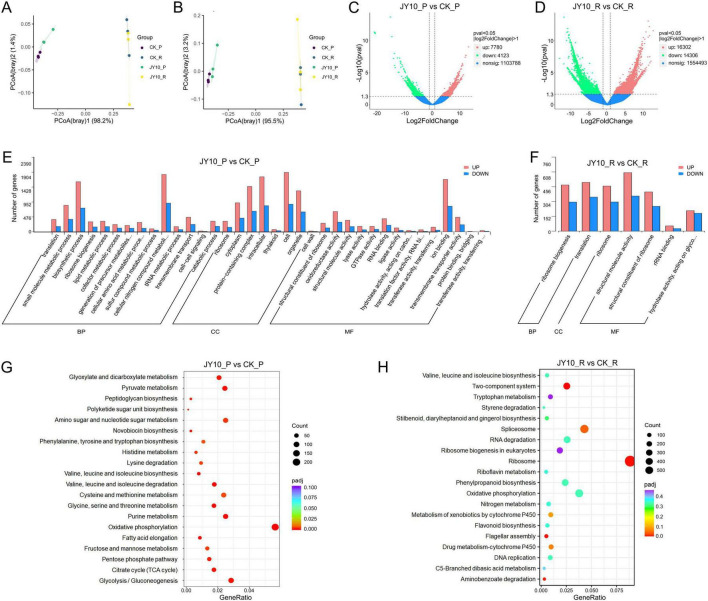
Functional category and analysis of DEGs (| log2 (Fold Change) | > 1 & *p*adj < 0.05). PCoA plot based on the eggNOG **(A)** and KO **(B)** category. Volcano plots for the DEGs between different groups (**C:** leaf, **D:** rhizosphere). GO functional classification (**E:** leaf, **F:** rhizosphere) and KEGG pathways enrichment analysis (**G:** leaf, **H:** rhizosphere) of DEGs in different comparison groups.

Finally, we employed metatranscriptomics to identify the core functional genes that were differentially expressed in the phyllosphere and rhizosphere of the host following JY10 treatment, concentrating on key metabolic pathways to elucidate the TTS control mechanisms of JY10. The metatranscriptomic sequencing generated a total of 85.58 Gbp of raw data, with 84.43 Gbp of clean data following quality control (Supplementary Table 7). The raw data were deposited in NCBI under bioproject submission number SUB15644262.The number of annotated genes was 844,494, 150,717, 1,048,575 and 1,045,753 by the GO, CAZy, eggnog, and KEGG databases, respectively. The eggNOG and KEGG functional categories of these differentially expressed genes (DEGs) of phyllosphere effectively distinguished between different samples, with samples within the same group clustering together. However, no significant differences were observed between the groups in any of the functional categories in the rhizosphere (Figures 6A,B). Compared to the control group, the DEGs in the phyllosphere and rhizosphere of JY10 treatment group were 11,903 and 30,608, respectively (Supplementary Table 8). Among them, 7,780 and 16,302 genes were upregulated, while 4,123 and 14,306 genes were downregulated, respectively (Figures 6C,D). In order to gain a deeper understanding of the potential functions of the DEGs following JY10 treatment, a GO functional classification analysis was conducted on the aforementioned DEGs. As illustrated in Figures 6E,F, the number of GO terms enriched in the phyllosphere exceeds that in the rhizosphere (Supplementary Table 9). In the biological process (BP) classification, the significantly enriched components identified in the phyllosphere included the cellular nitrogen compound metabolic process, biosynthetic process, small molecule metabolic process and translation. In contrast, the rhizosphere primarily exhibited enrichment in ribosome biogenesis and translation. In the classification of cellular components (CC), the phyllosphere showed significant enrichment in components such as cell, intracellular, protein-containing complex, organelle, cytoplasm and ribosome, while the rhizosphere was predominantly enriched in ribosome. Regarding the molecular function (MF) classification, the phyllosphere and rhizosphere both displayed significant enrichment in components such as structural constituent of ribosome, structural molecule activity, hydrolase activity, acting on carbon-nitrogen (but not peptide) bonds and RNA binding. The KEGG functional enrichment analysis revealed that the DEGs in the phyllosphere were significantly enriched in pathways related to oxidative phosphorylation, carbon metabolism, biosynthesis of secondary metabolites, biosynthesis of antibiotics, biosynthesis of amino acids, fatty acid metabolism, novobiocin biosynthesis (Figure 6G; Supplementary Table 10). In the rhizosphere, DEGs were mainly enriched in energy metabolism (including oxidative phosphorylation and nitrogen metabolism), xenobiotics biodegradation and metabolism (such as aminobenzoate degradation, drug metabolism-cytochrome P450, metabolism of xenobiotics by cytochrome P450 and styrene degradation), amino acid metabolism (including valine, leucine and isoleucine biosynthesis and tryptophan metabolism), biosynthesis of other secondary metabolites (such as stilbenoid, diarylheptanoid and gingerol biosynthesis, phenylpropanoid biosynthesis and flavonoid biosynthesis), flagellar assembly and quorum sensing. Among them, two-component system, ribosome, flagellar assembly, aminobenzoate degradation, and spliceosome were significantly enriched (Figure 6H; Supplementary Table 10). Notably, oxidative phosphorylation was identified as a representative pathway with a significant number of unigenes found in both phyllosphere and rhizosphere. Specifically, 228 DEGs related to oxidative phosphorylation were detected in phyllosphere, while 144 were found in rhizosphere. Among these, 166 DEGs in phyllosphere and 177 in rhizosphere were upregulated, whereas 62 DEGs in phyllosphere and 27 in rhizosphere were downregulated. Three DEGs, namely the F-type H+-transporting ATPase subunit, cytochrome c oxidase subunit II, and heme a synthase, exhibited a relatively high expression level (30–60-folds) in the marker genera of *Bacillus, Pseudonocardia*, and *Pseudomonas* identified in the metagenomics analysis. Conversely, heme o synthase demonstrated a downregulation trend across all three marker genera (Supplementary Table 8).

### 3.9 JY10 regulates antibiotics biosynthesis in tobacco plant

**FIGURE 7:**
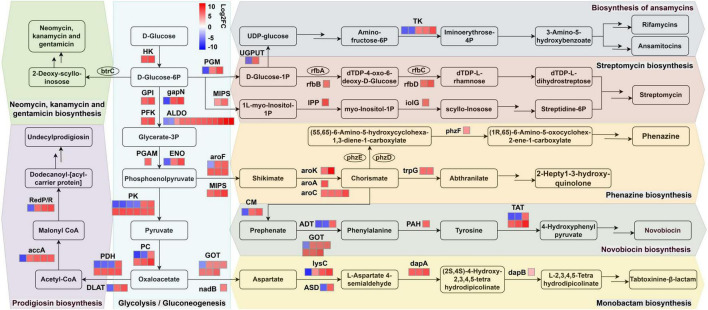
Schematic diagram of the main antibiotic biosynthesis pathways affected by DEGs in the JY10-vs.-CK comparison. Rectangles represent metabolites; Squares filled with colors and ellipses represent genes with and without significant changes, respectively. accA, acetyl-CoA carboxylase carboxyl transferase; ALDO, fructose-bisphosphate aldolase; ADT, arogenate/prephenate dehydratase; aroA, 3-phosphoshikimate 1-carboxyvinyltransferase; aroC, chorismate synthase; aroF, 3-deoxy-7-phosphoheptulonate synthase; aroK, shikimate kinase; ASD, aspartate-semialdehyde dehydrogenase; btrC, 2-deoxy-scyllo-inosose synthase; CM, chorismate mutase; dapA, 4-hydroxy-tetrahydrodipicolinate synthase; dapB, 4-hydroxy-tetrahydrodipicolinate reductase; DLAT, dihydrolipoamide acetyltransferase; ENO, enolase; gapN, glyceraldehyde-3-phosphate dehydrogenase; GOT, aspartate aminotransferase; GPI, glucose-6-phosphate isomerase; HK, hexokinase; iolG, myo-inositol 2-dehydrogenase; IPP, inositol-phosphate phosphatase; lysC, aspartate kinase; MIPS, myo-inositol-1-phosphate synthase; nadB, L-aspartate oxidase; PAH, phenylalanine-4-hydroxylase; PC, pyruvate carboxylase; PDH, pyruvate dehydrogenase; PFK, 6-phosphofructokinase; PGAM, 2;3-bisphosphoglycerate-dependent phosphoglycerate mutase; PGM, phosphoglucomutase; phzD, trans-2;3-dihydro-3-hydroxyanthranilic acid synthase; phzE, 2-amino-4-deoxychorismate synthase; phzF, phenazine biosynthesis protein; PK, pyruvate kinase; RedP/R, 3-oxoacyl-[acyl-carrier protein] reductase; rfbA, glucose-1-phosphate thymidylyltransferase; rfbB, dTDP-glucose 4;6-dehydratase; rfbC, dTDP-4-dehydrorhamnose 3,5-epimerase; rfbD, dTDP-4-dehydrorhamnose reductase; TAT, tyrosine aminotransferase; TK, transketolase; trpG, anthranilate synthase, UGPUT, UTP–glucose-1-phosphate uridylyltransferase.

We also noted that the biosynthesis pathways of some antibiotics, including novobiocin, monobactam, streptomycin, prodigiosin, phenazine, neomycin, kanamycin, gentamicin and ansamycins, were found to be regulated after JY10 treatment. Notably, the biosynthesis pathways of novobiocin (map00401), monobactam (map00261), and ansamycins (map01051) exhibited significant regulation (Figure 7; Supplementary Table 10). In the novobiocin biosynthesis pathway, five genes including chorismate mutase (*CM*), arogenate/prephenate dehydratase (*ADT*), aspartate aminotransferase (*GOT*), phenylalanine-4-hydroxylase (*PAH*) and tyrosine aminotransferase (*TAT*) showed significant changes. Except for *PAH*, where only one upregulated gene copy was detected, the other four genes exhibited copies from multiple species, with both upregulated and downregulated expressions observed among these copies. For most genes, the number and expression levels of the upregulated copies surpassed those of the downregulated ones, indicating that JY10 treatment enhanced novobiocin synthesis. Aspartate kinase (*lysC*), aspartate-semialdehyde dehydrogenase (*ASD*), 4-hydroxy-tetrahydrodipicolinate synthase (*dapA*), and 4-hydroxy-tetrahydrodipicolinate reductase (*dapB*) were the four sharply changed genes in the monobactam biosynthesis pathway. The expression levels of *dapA* and *dapB* were both upregulated, and the number of upregulated copies of *lysC* exceeded that of the downregulated ones, while *ASD* displayed an equal number of upregulated and downregulated copies and expression levels, indicating that JY10 treatment promoted monobactam synthesis. In the biosynthesis pathway of ansamycins, only one DEG named transketolase (*TK*) were detected, which had two downregulated copies and three upregulated copies. The overall upregulation expression level of *TK* is higher than the downregulation expression level, suggesting that JY10 treatment facilitated the biosynthesis of ansamycins through accumulating intermediate product iminoerythrose-4P. In other antibiotic biosynthesis pathways, the DEGs such as dTDP-glucose 4,6-dehydratase (*rfbB*), dTDP-4-dehydrorhamnose reductase (*rfbD*), inositol-phosphate phosphatase (*IPP*), and myo-inositol 2-dehydrogenase (*iolG*) in the streptomycin biosynthesis pathway, and DEGs such as3-phosphoshikimate 1-carboxyvinyltransferase (*aroA*), chorismate synthase (*aroC*), shikimate kinase (*aroK*), anthranilate synthase (*trpG*) and phenazine biosynthesis protein (*phzF*) in the phenazine biosynthesis pathway were predominantly upregulated. Similarly, most DEGs in the prodigiosin biosynthesis pathways were also upregulated. In contrast, the DEGs involved in the biosynthesis pathways of neomycin, kanamycin and gentamicin exhibited nonsignificant changes.

**FIGURE 8:**
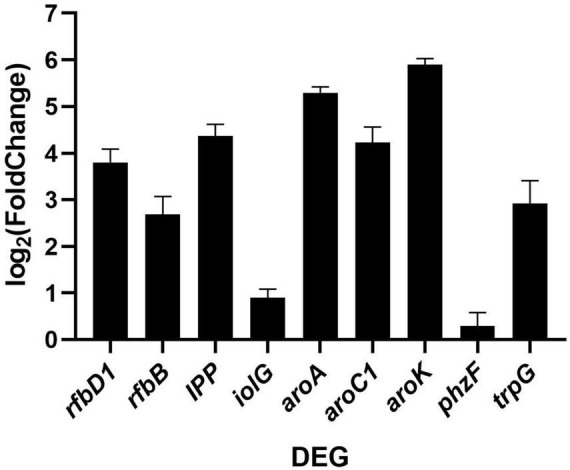
Gene expression validation of DEGs involved in streptomycin biosynthesis and benzene acid biosynthesis pathways. Fold change was estimated by the 2^– ΔΔCt^ method via qRT-PCR.

In order to validate the metatranscriptomic results, we conducted qRT-PCR to verify the DEGs involved in the streptomycin biosynthesis and phenazine biosynthesis pathways, as the DEGs in both pathways were all upregulated. As shown in Figure 8, in JY10_vs._CK comparison group, *rfbB, rfbD1* and *IPP* associated with streptomycin biosynthesis, as well as *aroA, aroC, aroK*, and *trpG* involved in phenazine biosynthesis were all significantly upregulated. In contrast, *iolG* and *phzF* in these two pathways did not show significant changes (| log2 (Fold Change) | < 1), indicating that the qRT-PCR results closely aligned with the trends observed in the metatranscriptomic data. These findings suggested that JY10 treatment enhanced the biosynthesis of novobiocin, monobactam, streptomycin, prodigiosin, phenazine, and ansamycins, thereby conferring resistance to pathogens in tobacco.

## 4 Discussion

*Bacillus velezensis* has emerged as a promising biocontrol agent in agriculture due to its broad-spectrum antagonistic activity against various plant pathogens and its ability to promote plant growth (Keshmirshekan et al., 2024; Kim et al., 2022). For instance, *B. velezensis* BV01 showed inhibitory rates against phytopathogens such as *Bipolaris sorokiniana, Botrytis cinerea, Colletotrichum capsici, Fusarium graminearum, Fusarium oxysporum, Neocosmospora rubicola, Rhizoctonia solani*, and *Verticillium dahlia* ranging from 36 to 92%, demonstrating significant control effects on wheat root rot and pepper Fusarium wilt in greenhouse (Huang et al., 2023). Furthermore, *B. velezensis* strains BZR 336 and BZR 517 isolated from southern Russia have been shown to effectively control potato stem canker and black scurf diseases caused by *R. solani*, with a reduction in disease incidence of up to 93.5% (Asaturova et al., 2021). A recent study revealed that *B. velezensis* FZB42, known for its ability to promote plant growth, effectively colonize the rhizosphere, and exhibit a broad spectrum of antagonistic activities against phytopathogens, thereby improving the resistance of rice plants to sheath blight disease caused by *R. solani* (Ali et al., 2025). However, there are no reports about the utilization of *B. velezensis* for the biocontrol of TTS disease caused by *R. solani.* This study identifies a strain of *B. velezensis* JY10, which not only exhibits significant inhibitory effects against *R. solani* but also enhances the resistance of tobacco plants to TTS disease caused by *R. solani* in pot experiments, indicating that JY10 has potential as a green agent for the control of TTS.

JY10 was identified as *B. velezensis* based on two facts. Firstly, morphological observation revealed that it is a Gram-positive bacterium with colonies having a smooth and flat surface and neat edges, consistent with the typical characteristics of *Bacillus* genus (Whitman, 2010). Additionally, the physiological and biochemical properties of JY10 are in line with those of *B*. *velezensis*, although some differences are noted (Guo et al., 2020). For example, JY10 can utilize D-maltose and α-lactose but not D-fructose, while *B. velezensis* XF-8 can utilize D-fructose but not D-maltose or α-lactose (Xu et al., 2025). Secondly, comparative genomic analysis confirmed that JY10 is affiliated to *B. velezensis.* Recent advancements in phylogenetic construction based on core genomes have contributed to a more standardized bacterial taxonomy (Parks et al., 2018). ANI is the average identity value derived from pairwise comparisons of homologous sequences between two genomes, which is commonly employed in species definition (Lee et al., 2016). In this study, a phylogenetic tree of 18 *Bacillus* genomes was constructed based on the concatenation of 560 single-copy core genes present in all genomes using the ML method. Additionally, a heatmap analysis based on the ANI values of these *Bacillus* strains confirmed that JY10 is indeed affiliated with *B. velezensis*.

In addition to being used for strain identification, the whole genome sequences of *B. velezensis* provide researchers with a comprehensive understanding of the strain’s role, thereby guiding the development and applications of *B. velezensi*s (Su et al., 2024). Numerous studies have demonstrated that *B. velezensi*s strains exert biocontrol efficacy through secreting active secondary metabolites, such as cyclic lipopeptides (bacillibactin, bacillomycin-D, fengycin, iturin, mycosubtilin, plipastatin, surfactin), polyketides (bacillaene, difficidin, macrolactin), other peptides (amylocyclicin, bacilysin, butirosin, ericin, plantazolicin), and volatile organic compounds (VOCs) (benzothiazole, 4-chloro-3-methyl-phenol, 2,5-dimethl-pyrazine, and 2,4-bis (1,1-dimethylethyl)-phenol) (Gao et al., 2017; Keshmirshekan et al., 2024; Rabbee et al., 2019). Among these metabolites, bacilysin, amylocyclicin, ericin, macrolactin, bacillaene, difficidin, and mycosubtilin exhibited antibacterial activities, while iturin, fengycin, and plipastatin demonstrated antifungal properties. Additionally, bacillibactin played a crucial role in facilitating the acquisition of ferric ions (Fe^3+^) from minerals and organic compounds in the rhizosphere, inhibiting the growth of phytopathogens by depriving them of essential iron ions (Rabbee et al., 2019). Similar to many *B. velezensi*s strains, JY10 exerted its antimicrobial effect by synthesizing surfactin, fengycin, macrolactin H, bacillaene, difficidin, bacilysin, and bacillibactin, but it could not synthesize amylocyclicin, ericin, or iturin. Furthermore, JY10 could synthesize an anti-bacterial agent butirosin A/B, which could not be synthesizing by *B. velezensi*s ES2-4, E68 TSA32-1, 157 and 9D-6 (Keshmirshekan et al., 2024).

It is well established that beneficial microbes can compete with pathogens for ecological niches, alter the structure of microbial communities, recruit beneficial taxa, and suppress pathogens in residues, thereby protecting the host from pathogenic invasion (Jin et al., 2025, Köhl et al., 2019, Zeng et al., 2025, Zhang et al., 2025). However, based on β-diversity analysis, the JY10 treatment did not significantly alter the microbial community structure in the leaves and roots. The reason for this may be that JY10 originally colonized the stems, and when sprayed onto the leaves, the high ultraviolet radiation, rapid drying, and unstable microclimate of the leaves limited its sustained rapid reproduction (Bashir et al., 2022). Another possible reason was the priority effect and competitive exclusion effect of the indigenous microbial community on the leaves. After the application of exogenous microorganisms, JY10 faced resource competition from the local flora, which made it less dominant in the short term and thus unable to significantly change the microbial community composition of the leaves (Sohrabi et al., 2023, Vorholt, 2012). Additionally, JY10 was applied as a foliar spray, colonizing the leaves without migrating to the roots, and therefore had minimal impact on the microbial community composition of the rhizosphere. Although JY10 had a minor impact on the structure of the host microbial community, it significantly altered the abundance of several specific microorganisms. Further LEfSe analyses indicated that it substantially increases the abundance of *Bacillus, Pseudonocardia*, and *Pseudomonas*. The genus *Pseudonocardia*, a member of Actinomycetales, can generate bioactive secondary metabolites, including variably glycosylated polyenes and antibiotics (Whatmough et al., 2024). It exerts its anti-bacterial and anti-fungal activities by producing active compounds such as 4-(2-acetamidoethyl) phenyl acetate, 4-((1,4- dioxo-octahydro-pyrrolo [1,2-a] pyrazin-3-yl) methyl) phenyl acetate, pseudonocardians, and garamycin (Riahi et al., 2022). *Pseudomonas*, including *P. chlororaphis, P. fluorescens, P. putida* and *P. protegens* have been successfully identified and proven effective in controlling plant diseases and promoting plant growth. They not only produce antibiotics and other secondary metabolites that suppress pathogen growth, but also colonize plant roots and form a protective barrier against pathogenic invasion (Santoyo et al., 2025, Venturi, 2022). The increase in the abundance of these beneficial bacteria helps to enhance the TTS disease-resistance ability of tobacco. However, it is important to note that our experiment was not conducted under sterile conditions, and exogenous microorganisms could interfere with our results. Therefore, a more stringent sterile system, point-to-point experiments (coculture JY10 with a single beneficial bacterium), or synthetic microbial community experiments (coculture JY10 with a set of beneficial bacteria) were required to verify whether JY10 treatment actually increased the abundances of these beneficial microorganisms.

We further utilized the metatranscriptomics to investigate the mechanism by which JY10 controls TTS disease by identifying the changes in the gene expression of microorganisms in the tobacco phyllosphere and rhizosphere following JY10 treatment. Metatranscriptomics provided a practical approach to uncovering the intrinsic microscale mechanisms underlying the ecological roles of microorganisms beyond mere pathway presence (Yan et al., 2024). After treatment with JY10, the expression levels of various genes within the same species in both the phyllosphere and rhizosphere regions of tobacco, as well as the expression of identical genes across different species, exhibited significant alterations. Most of these genes were enriched in pathways such as oxidative phosphorylation, valine, leucine and isoleucine biosynthesis, ribosome, nitrogen metabolism, and C5-Branched dibasic acid metabolism. Notably, oxidative phosphorylation emerged as the pathway with the most significant changes, involving the greatest number of genes. To thrive, cells must rapidly respond to changes in metabolic demand and environmental conditions. In particular, a cell must possess the ability to modulate oxidative phosphorylation and ATP production to ensure an adequate energy supply, especially when the host plant is subjected to pathogen invasion. The modulation of oxidative phosphorylation is known to play a crucial role during developmental processes in both prokaryotes and eukaryotes (Magalon and Alberge, 2016). This study found that the expression of most DEGs involved in oxygen detoxification was upregulated after JY10 treatment, suggesting that JY10 might indirectly enhance the resistance of tobacco to TTS disease by modulating the oxygen phosphorylation of the host microbiome. This finding was similar to the report that oxidative phosphorylation and glycerol-1-phosphatase in the rhizosphere might play critical roles in enhancing Chinese cabbage’s resistance to clubroot disease caused by *Plasmodiophora brassicae* Woron (Liu et al., 2024). Interestingly, in animal husbandry, *B. subtilis* had been shown to protect ducks from *Escherichia coli* infection through upregulating oxidative phosphorylation complex genes and restoring mitochondrial function (Li et al., 2022), indicating that the regulation of oxidative phosphorylation may positively influence disease control.

In addition to the oxidative phosphorylation pathway, the biosynthetic pathways of various antibiotics, including novobiocin, monobactam, streptomycin, prodigiosin, phenazine, neomycin, kanamycin, gentamicin, and ansamycin, were also regulated following JY10 treatment. Most of DEGs involved in these pathways being upregulated, and our qRT-PCR results corroborated this finding that DEGs *rfbB, rfbD1 IPP*, *aroA, aroC, aroK*, and *trpG* involved in streptomycin biosynthesis and phenazine biosynthesis pathways were all significantly upregulated. Among these antibiotics, novobiocin, monobactam, streptomycin, neomycin, kanamycin, gentamicin, and ansamycin exhibit broad-spectrum antimicrobial activity. Prodigiosin, a microbial alkaloid red pigment characterized by a tripyrrole structure, possesses antibacterial, antifungal, and algicidal activities (Islan et al., 2022). Phenazine functions as an electron shuttle that can modulate the cellular redox state and influence the expression of downstream genes related to biofilm formation and bacterial survival. Furthermore, phenazine serves as a biological control agent that can affect plant growth and induce systemic resistance in plants (Salwan and Sharma, 2020).

Overall, JY10 not only has the potential to secrete antifungal metabolites autonomously but may also enhance the synthesis of antibiotics by other microorganisms present in the tobacco phyllosphere and rhizosphere, thereby improving the tobacco’s resistance to TTS disease. However, it must be noted that these results were derived from *in silico* predictions and speculation based on omics. Further experimental verification is required to determine whether JY10 itself can produce antibiotics and whether it can enhance the production of antibiotics by other microorganisms. We planned to purify the main antifungal compounds of JY10 after it had been successfully used to effectively control TTS in field trials for at least 2 years.

## 5 Conclusion

In this study, we isolated a bacteria strain JY10 with potent anti-*R. solani* activity from healthy tobacco leaves. Additionally, JY10 demonstrated strong inhibitory effects on *C. fructicola* and *B. linicola*, the pathogens responsible for tobacco anthracnose and tobacco Phoma leaf spot disease, respectively. Moreover, JY10 achieved a TTS control effect of up to 68.63% in pot experiments. Based on the morphological, physiobiochemical and genomic characteristics, the strain JY10 was identified as *B*. *velezensis*. AntiSMASH analysis predicted 12 secondary metabolite biosynthetic gene clusters, encoding antimicrobial compounds such as surfactin, fengycin, difficidin, bacillaene, bacillibactin, macrolactin H, and bacilysin, potentially responsible for the inhibition of *R. solani*. Treatment with JY10 did not significantly alter the microbial community structure in both the tobacco phyllosphere and rhizosphere, as indicated by alpha diversity analysis, but it significantly increased the relative abundance of beneficial microorganisms, including *Bacillus*, *Pseudonocardia*, and *Pseudomonas.* Furthermore, Metatranscriptomics profiling showed that JY10 might endow tobacco TTS resistance by modulating oxidative phosphorylation and upregulating antibiotic biosynthesis pathways, such as novobiocin, monobactam, streptomycin, prodigiosin, phenazine, neomycin, kanamycin, gentamicin and ansamycins biosynthesis pathways. These new findings open possible applications for JY10 in the green control of TTS in field.

## Data Availability

The datasets presented in this study can be found in online repositories. The names of the repository/repositories and accession number(s) can be found in this article/Supplementary material.

## Footnotes

1
http://jspecies.ribohost.com/jspeciesws/

2
https://github.com/cjfields/readfq

3
https://github.com/OpenGene/fastp

4
http://bowtie-bio.sourceforge.net/bowtie2/index.shtml

5
https://github.com/voutcn/megahit

6
http://topaz.gatech.edu/GeneMark/

7
http://www.bioinformatics.org/cd-hit/

8
https://www.ncbi.nlm.nih.gov/

9
http://www.ncbi.nlm.nih.gov/COG/

10
http://www.kegg.jp/kegg/

11
http://eggnog5.embl.de/#/app/home

12
http://www.cazy.org/

13
https://github.com/bbuchfink/diamond/

14
https://github.com/trinityrnaseq/trinityrnaseq/wiki

15
https://github.com/Oshlack/Corset/wiki
